# The Role of Hunters in Wildlife Health Research and Monitoring: Their Contribution as Citizen Scientists in Italy

**DOI:** 10.3390/ani14152204

**Published:** 2024-07-29

**Authors:** Stefania Zanet, Francesco Benatti, Manuela Poncina, Carlotta Pasetto, Mario Chiari, Michele Sorrenti, Ezio Ferroglio

**Affiliations:** 1 Dipartimento di Scienze Veterinarie, Università degli Studi di Torino, Largo Braccini 2, 10095 Grugliasco, Italy; 2 Federazione Italiana della Caccia, Via Garigliano 57, 00198 Roma, Italy

**Keywords:** wildlife, surveillance, monitoring, hunting, citizen science

## Abstract

**Simple Summary:**

Emerging and re-emerging infectious diseases are a significant burden on the global economy, public, and animal health. These events are predominantly zoonotic (60.3%), with the majority (75%) originating from wildlife. In Italy, there are hundreds of research, monitoring, and surveillance activities targeting wildlife-related pathogens. The objective of this review is to highlight the importance of hunters in the context of disease research and surveillance in wild populations. A total of 192 papers, 16 theses, and 94 congress abstracts were selected by applying a specific string and inclusion criteria. The contribution of hunters amounts to nearly 400,000 sampled animals. These results underscore the paradigm of One Health in wildlife health surveillance, emphasizing the crucial role of hunters in sample availability, which forms the foundation of current health surveillance activities.

**Abstract:**

In Italy, there are hundreds of research, monitoring, and surveillance activities targeting emerging and re-emerging pathogens. These activities heavily rely on hunters for sample collection and early identification of morbidity/mortality events. The objective of this review is to describe and quantify the contribution of hunters in the context of disease research, monitoring, and surveillance in wild populations. A literature review and descriptive summary statistics were performed following PRISMA-2020 guidelines; articles were obtained from major scientific databases, abstracts from national and international conferences, proceedings, graduate-level theses from online library repositories, and direct contact with academic experts. The contribution of hunters in terms of sample collection for health-related activities on wildlife amounts to 400,000 sampled animals. Wild boars were involved in 158 surveillance systems/research studies, followed by red deer (71), foxes (63), and roe deer (59). The pathogens under surveillance were mainly zoonotic (Salmonella spp.), emerging (Hepatitis E virus), and/or vector-borne (West Nile virus). The temporal distribution of scientific papers followed a positive trend that reflects the growing interest in wildlife from different sectors. These results highlight how wildlife health-related efforts are a paradigm of the concept of One Health, in which the role of hunters is crucial to ensure sample availability, and it constitutes the base of much current wildlife health research, monitoring, and surveillance.

## 1. Introduction

Emerging infectious diseases (EIDs) are a significant burden on global economies and public health. EID events are dominated by zoonoses (60.3% of EIDs); the majority of these (71.8%) originate in wildlife and have increased significantly over time [1]. Despite the primary role of wildlife in the epidemiology and evolution of EIDs [2], the surveillance, monitoring, and collection of wildlife-related data are often misrepresented [3]. A recent survey was carried out among European Union (EU) member states and neighboring countries, aimed at describing and mapping the main existing structured and systematic initiatives for the surveillance of zoonotic, transboundary, or emerging pathogens that involve wildlife. Passive surveillance resulted as the most frequently applied sampling strategy for wildlife among the EU surveillance systems (SSs). It is applied as the sole method of sampling in 60% of SSs or in association with active collection sampling in 31.1%. Game species are the most frequently targeted by both active and passive surveillance activities, with game mammals being targeted in 16.9% and game birds in 6.7% of the SS. *Brucella* spp., Influenza A virus, Rabies virus, *Echinococcus* spp., *Francisella tularensis*, West Nile virus, *Coxiella burnetii*, *Leptospira* spp., Tick-borne encephalitis virus, *Toxoplasma gondii*, and Hepatitis E virus are the most frequently targeted pathogens [4].

In Italy, wildlife-related pathogen surveillance is highly heterogeneous, varies geographically, and is managed mostly at the regional or subregional level (Nomenclature of Territorial Units for Statistics—NUT2 or NUT3). Surveillance plans differ locally in organization, extent of sampling, targeted species, and samples’ destination and processing. Today, more than 15 pathogens are targeted by either national or sub-national surveillance plans [5,6,7,8,9,10], including Avian and Swine Influenza, Rabies, Tuberculosis, West Nile and Usutu Virus, *Echinococcus* spp., and African and Classical Swine Fever Virus. At the same time, a great effort in terms of research is carried out nationwide every year by researchers working within universities or Experimental Zooprophylactic Institutes, as demonstrated by the number of sources included in this review. Within this heterogeneous organization, systematic and structured surveillance systems, as well as individual research studies, heavily rely on hunters for sample collection, population observation, and monitoring. In this perspective, hunters, while carrying out recreational activities or by performing culling activities for population control and management, provide an essential service not only for wildlife population management and control but also for veterinary and public health. For the purpose of this work, hunters are defined as individuals duly authorized and compliant with legal regulations to hunt specific animal species under precisely defined conditions, as stipulated by law. Under certain conditions regulated by law, hunters also carry out culling activities for population management and control. The role of hunters as citizen scientists has long been recognized as they contribute to wildlife management by providing hunting bag data, observations for population counts and indices [11], and are also engaged in population control of invasive or overabundant species [12]. The goal of this paper is to emphasize, through the use of a systematic review and ‘descriptive summary statistics’ approach, their active and essential role in novel aspects of wildlife management, which are researching, monitoring, and surveilling wildlife-related pathogens.

## 2. Materials and Methods

We carried out a systematic review and descriptive summary statistics of the scientific literature concerning the role of hunters in research, monitoring, and/or surveillance activities targeting wildlife-related pathogens in Italy. To build our protocol, we adhered to the specific items of the checklist for systematic reviews (PRISMA-P 2020) [13]. Scientific studies to be included in the analysis were retrieved from different sources, as reported in Table 1.

For the systematic interrogation of scientific literature databases, the following string was applied after being previously validated to maximize sensitivity and precision:

(monitor* OR prevalence OR epidemiology OR surveillance OR disease* OR pathogen* OR health) AND (wild* OR bird* OR deer OR boar OR fox OR mouflon OR chamois OR waterfowl OR partridge OR grouse OR hare OR rabbit OR lagomorph OR cottontail) AND (ital*) AND (hunt* OR cull*)

Academics were asked to report the eligible theses of undergraduate and graduate students, as well as the gray or unpublished eligible literature. The indexed papers, graduate theses, and gray literature sources covered a time span from 2000 to July 2022, while conference abstracts were considered starting from 2015 onwards. The different time intervals are justified by the availability of sources; in fact, articles and theses were filtered and requested from experts from 2000 to 2022, considering the increased emphasis in recent years on the topic. With regard to abstracts, the time interval covers the period from 2015 to 2022, since abstract books of the previously identified target conferences/congresses were available online only for this time period.

The results of the literature search were collected and analyzed by constructing a dedicated data model. After removing duplicates, the full text of target conferences’ abstract books and thesis repositories were fully consulted and screened for pertinent content according to the subsequent inclusion criteria: i. presentation of novel eco-epidemiological data on homeothermic fauna, ii. samples originating from hunting/control activity. A literature search was performed by three independent reviewers (F.B., M.P., and C.P.), and disagreements between reviewers were resolved by a fourth author (S.Z.), consulted when necessary.

A list of eligible studies for data extraction was managed in Microsoft Excel. A data extraction sheet was created in Microsoft Excel. F.B., M.P., and C.P. extracted data from eligible studies. S.Z. double-checked the quality of data extraction. The following information was collected for each study: ID; sources; references; abstract; link; DOI; publication type; objective; region (NUT2 level); province (NUT3 level); origin (population control plan or recreational hunting activity); type of surveillance (active or passive); organization; year(s); duration; hunting seasons; species; sample type (tissue/organ collected); metadata (anagraphical data, biometrical data, georeferencing); type of analysis (direct analysis, such as parasitological—copromicroscopic exam; bacteriological—bacterial culture; virological—viral isolation; molecular biology—PCR; macroscopic analysis—necropsy, chemical and physical analysis; microscopic analysis—cytology and histopathology); indirect analysis—ELISA, complement fixation test; pathogens and environmental contaminants; sector involved; collaboration; main outcome/conclusions; institutions; and pertinent references. The full data model used for analysis is available in Annex S1.

To minimize the risk of bias, the authors noted any information that could affect the interpretation of the articles’ contents. Outliers were investigated using leave-one-out analysis [39]. After completing the data model with the information from the included bibliographic sources, descriptive, quantitative, and qualitative analyses were carried out to assess hunters’ involvement in surveillance systems with particular emphasis on geographical involvement, hunting species, target pathogens, and temporal distribution. For each category, the total number of sources (299) in which they were found and the percentage of total sources were calculated considering that the same source could report more than one category at the same time (e.g., objectives, conclusion, regions). All analyses were carried out using R version 3.4.0 [40].

## 3. Results

**Figure 1:**
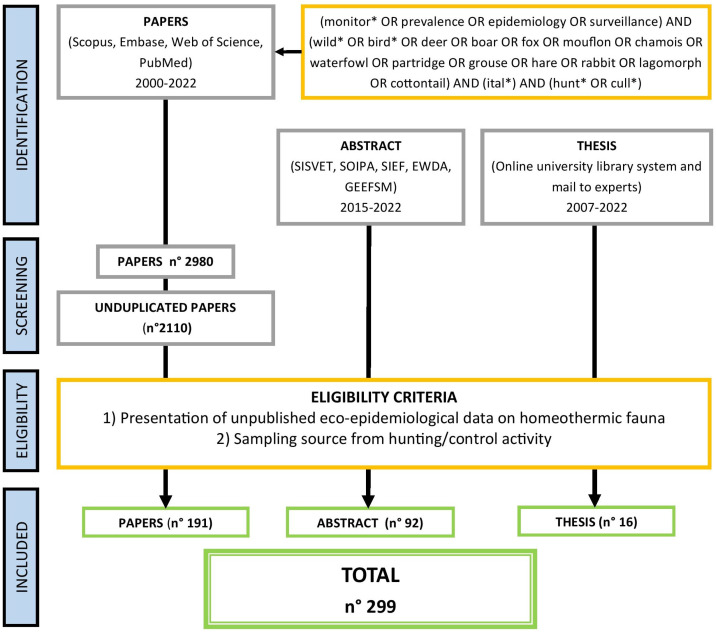
Flowchart showing the process that led to the source selection subsequently analyzed.

A total of 2980 peer-reviewed articles were extracted from the selected databases (Table 1). After duplicates’ removal, 2110 were screened on the basis of inclusion criteria and 191 were actually included in the ‘descriptive summary statistics’. In addition, 16 graduate theses and 92 congress abstracts were included (Figure 1).

A total of 299 studies satisfied both inclusion criteria and underwent complete analysis. The full data model with raw data for the 299 studies is available as Supplementary Material (Annex S1).

**Figure 2:**
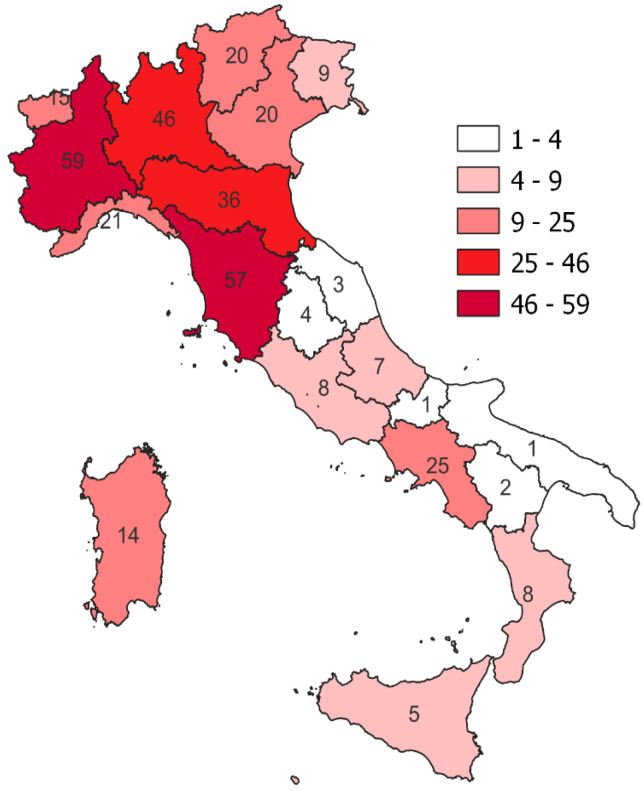
Geographical distribution of wildlife health surveillance studies involving hunters.

Geographical distribution of studies reporting hunters’ involvement is depicted in Figure 2. The highest contribution of hunters to wildlife disease surveillance activities and research was recorded in the Piedmont (*n* = 59, 19.7%), Tuscany (*n* = 57, 19%), Lombardy (*n* = 46, 15.4%), and Emilia-Romagna (*n* = 36, 12%) regions.

Other territories such as Umbria (*n* = 4, 1.3%), Marche (*n* = 3, 1%), Basilicata (*n* = 2, 0.7%), Apulia (*n* = 1, 0.3%), and Molise (*n* = 1, 0.3%) reported a lower involvement.

The species most frequently sampled is wild boar, with a total of 294,903 animals sampled in 153 studies (51.2%). Notably, wild boar is also the most heavily hunted species, together with roe deer at the national and European levels [41]. The other species sampled, the number and percentage of total sources where they are sampled, and the total number of animals sampled are shown in Table 2.

**Figure 3:**
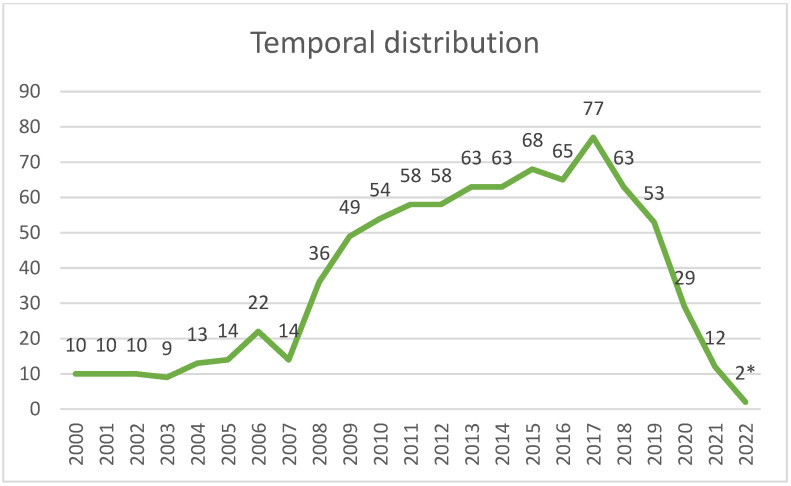
Temporal distribution of wildlife health-related studies involving hunters. * The search was conducted from July 2022, so the number of sources is incomplete and underestimated for the same year.

The temporal distribution denotes a steady increase since 2008, which peaked in 2017 and has since then registered a negative trend (Figure 3).

Research, monitoring, and surveillance activities on wildlife-related pathogens in which hunters are involved respond to specific goals. One or more aims have been identified for each article, as shown in Table 3.

The tissues/organs most frequently sampled from hunted animals are shown in Table 4. In addition to these, other less frequently collected samples are central nervous system (*n* = 26, 8.7%), urinary bladder (*n* = 6, 2.0%), ectoparasite (*n* = 22, 7.4%), portion of skin (*n* = 20, 6.7%), ear (*n* = 4, 1.3%), trachea (*n* = 7, 2.3%), male reproductive tract (*n* = 8, 2.7%), female reproductive tract (*n* = 7, 2.3%), fetus and placenta (*n* = 8, 2.7%), bone marrow (*n* = 2, 0.7%), tongue (*n* = 4, 1.3%), and diaphragm (*n* = 14, 4.7%) samples.

Within the selected sources, some essential metadata (age and sex, biometrics and weight of hunted animals, geographical location) obtained during health inspection of hunted wildlife or directly collected by hunters were recorded (Table 4). The analytical methods used to analyze the sampled tissues were summarized into direct (molecular biology, parasitological analysis, bacteriological analysis, viral isolation, gross pathology, cytological and histopathological analysis, chemical and physical analysis) and indirect methods (e.g., ELISA, complement fixation test) [4] (Table 4). The frequency with which they were applied is reported in Table 4. A total of 115 bacterial, viral, parasitic, contaminant, and AMR strains were targeted in the selected studies. The most frequently targeted are reported in Table 5, while the complete list is presented in Annex S2.

**Figure 4:**
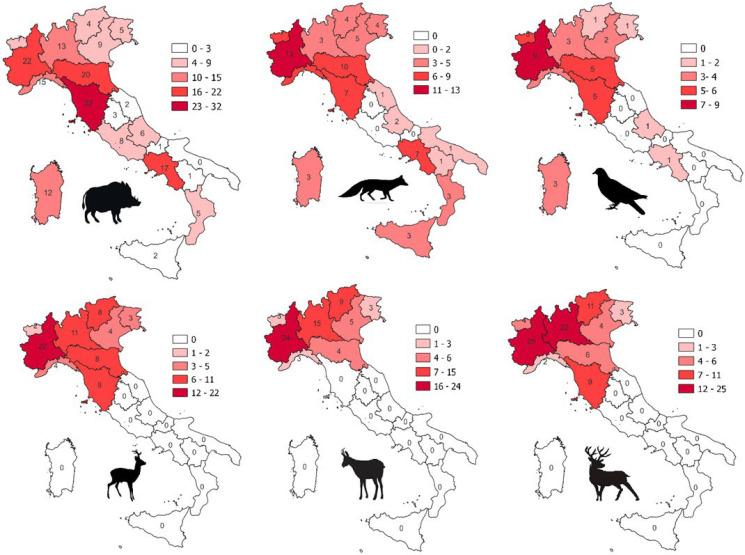
Geographical distribution of studies carried out in each region of Italy on the main hunted species (wild boar, red deer, red fox, wild birds, roe deer, and chamois).

The different sectors involved in the considered research/monitoring/surveillance activities and the institutions in charge of the organization and coordination of the studies are shown in Table 3. The regions were also related to the species most sampled in the studies analyzed (Figure 4).

Each bibliographic source identified one or more outputs and outcomes, which are listed in Table 3. No relevant comments were identified by the reviewers that could have affected the outcomes of the studies included.

## 4. Discussion

Traditionally, the role of hunters has been evaluated in relation to their contribution to wildlife population management [11]. However, it has been shown that they can also play an important role in wildlife disease detection and epidemiological surveillance [42,43]. In contexts where resources are limited and cost-effectiveness is paramount, hunters and citizens at large, are valuable resources that can also be of paramount importance when surveillance systems evolve from opportunistic surveillance to structured and harmonized schemes [42].

In particular, this is the first systematic review and descriptive summary statistics focused on assessing hunters’ contributions to wildlife health surveillance in Italy. Italy was chosen as a case study due to the high variety of hunting systems, hunted and culled species, and the plurality of wildlife surveillance/monitoring and research efforts at the national level. The results concerning the geographical distribution of the studies included in our work mirrored regional differences in hunting activities and hunting management. The geographical disparity that emerged from the data can be related to the presence of game control centers in the territory (especially in central and northern Italy) and to the type and number of species that are predominantly hunted in the various regions. Wildlife control centers, thanks to the presence of ad hoc trained staff, play a fundamental role in the management of wild populations (demographic and biometric evaluation of hunted animals). In addition, they perform a fundamental role as epidemiological observation points for wildlife diseases by evaluating all animals culled within their respective territories. At the same time, they act as an interface between public institutions, universities, and IZSs, which organize and coordinate both research and surveillance activities and hunters.

Relative to the number of species hunted in the territory, our data underlie how the number and distribution of analyzed species within the national territory are not uniform. In southern regions, hunting and culling activities are mainly focused on wild boar, red foxes, and birds; the latter are widely present across the national territory with differences in species presence and abundance related to local bioecological conditions, but surveillance and research activities are concentrated in regions of northern Italy, where the epidemiological interest is driven by the local presence of intensive farming (i.e., poultry and swine in Lombardy, Emilia-Romagna, Veneto, and Piedmont or cattle in Lombardy, Veneto, and Piedmont). The distribution and abundance of ruminant species are variable within the national territory, and this is partially reflected in the surveillance and research efforts that emerged from our data. Ruminants are present with a higher number of species and with greater abundance in northern alpine areas and in the Apennines [44].

A visualization of the temporal distribution of the collected studies showed a clear increase from 2008 onwards, with a drastic reduction from 2020 to the present, most probably related to the SARS-CoV-2 pandemic which catalyzed attention and funding. Conversely, the 2008-onward increase confirms the more general trend of raising awareness of the growing numbers of free-ranging wildlife populations and human-induced biotic and abiotic changes in the environment, livestock-raising practices which contribute to creating favorable circumstances for pathogen maintenance and spread at the human–livestock–wildlife interface [45,46].

Regarding the metadata collected, georeferencing information at various levels of precision is the most frequently reported data, which is fundamental for correct disease prevention and management [46]. Higher precision levels, which report either the location of culling at the municipality level (LAU2—Local Administrative Units level 2) or the exact coordinates where the animal was collected, are limited to those hunting systems managed by wildlife control centers. Once again, wildlife control centers, thanks to the presence of trained personnel, are essential not only for population management (i.e., collecting biometric data used to assist management decisions) but also for wildlife disease prevention and control, serving as an on-the-field observation standpoint, thus allowing an effective assessment of diseases’ spatio-temporal dynamics [47].

Intracardiac and abdominal clots were the most frequently collected samples. This biological matrix is particularly easy to identify and collect by hunters and can be used for a variety of molecular and serological diagnostics. Among the analytical methods, the one most widely implemented is PCR because of its sensitivity and versatility of use for multiple matrices and pathogens.

Overall, hunters’ involvement encompassed 115 pathogens and contaminants. Zoonotic pathogens and particularly food-borne pathogens (i.e., *Salmonella* spp., *Yersinia enterocolitica,* Hepatitis E virus, *Trichinella* spp., *Toxoplasma gondii*) are the ones most frequently targeted (*n* = 76, 66.1%). The hot topic of antimicrobial resistance was also targeted by *n*= 13 literature sources (11.3%), while the most targeted contaminants were ochratoxin A, whose presence is related to global climate change [48], heavy metals, polychlorinated biphenyls, perfluorinated alkylated substances, polybrominated diphenyl ethers, organochlorine, and organophosphorus, which are widespread due to agricultural use and industrial pollution [49]. This work highlighted profound differences related to hunting practices, local wildlife management, and the interest and publication preferences of the researchers involved. On the other hand, despite these differences, the goals and results obtained by the analyzed literature sources showed a homogeneous overlap. Among the most frequently proposed objectives were the acquisition of epidemiological data (66.6%) and pathogen identification (22.7%), while prevalence estimation (64.5%) and assessment of the endemicity and reservoir role of wild species (34.4%) were the most commonly obtained outcomes.

## 5. Conclusions

Overall, from the literature sources included in this study, hunters provided nearly 400,000 animals for the surveillance and research of 115 pathogens and contaminants. These numbers highlight and confirm the role of hunters in the monitoring and management of wildlife diseases, as reported in previous studies [42,43]. In the world of open science, open data, and crowdsourcing, hunters can be considered as real forerunners as they have been citizen scientists long before this concept was commonly applied. Traditionally, hunters have contributed to wildlife management by providing hunting bag data, observations for population counts and indices [10], and are engagement in population control of invasive or overabundant species [11].

## Figures and Tables

**Table 1:** Sources used to retrieve scientific studies for descriptive summary statistics.

Scientific Literature Databases (2000–2022)	Format
Embase, Pub Med, Scopus, Web of Science	Online
Abstract books of national and international conferences (2015–2022)	
SISVET (Italian Society of Veterinary Sciences) 2015, 2016, 2017, 2018, 2019, 2021	Printed and online [14,15,16,17,18,19]
SOIPA (Parasitological Italian Society) XXVII, XXVIII, XIX, XXX, XXXI, XXXII	Printed and online [20,21,22,23,24,25]
SIEF (Italian Society of Wildlife Ecopathology) 2017, 2019	Online [26,27]
EWDA (European Wildlife Disease Association) 2018, 2021	Online [28,29]
GEEFSM (Groupe d’Etudes sur l’Ecopathologie de la Faune Sauvage de Montagne) 2016, 2017, 2018, 2019, 2021	Online [30,31,32,33,34]
Master and/or doctoral thesis repository (2005–2022)	
Library of Agricultural and Veterinary Sciences—University of Torino	Online [35]
University Library System, University of Pisa	Online [36]
Institutional doctoral theses repository—University of Bologna	Online [37]
Open Archive—University of Naples, Federico II	Online [38]
Direct contacts via mail to academics and researchers operating in Italy who actively work on target topics	

**Table 2:** Table reports the total number of animals sampled for each species/taxa considered, along with the number of bibliographic sources (and %) where the species/taxa were included.

Species	*n*. Sampled Animals	*n*. of Reporting Sources (%)
Wild Boar (*Sus scrofa*)	294,903	153 (51.2)
Bird (*Aves*)	33,285	31 (10.4)
Red Fox (*Vulpes vulpes*)	26,637	63 (21.1)
Red deer (*Cervus elaphus*)	17,937	72 (24.1)
Chamois (*Rupicapra rupicapra*)	10,082	52 (17.4)
Roe deer (*Capreolus capreolus*)	8709	59 (19.7)
Fallow deer (*Dama dama*)	3012	18 (6.0)
Lagomorph (*Lagomorpha*)	2331	19 (6.4)
Mouflon (*Ovis g. musimon*)	259	10 (3.3)
Coypus (*Myocastor coypus*)	108	2 (0.7)

**Table 3:** The table shows the percentage and absolute number of bibliographical sources that detail the aims and scopes of surveillance, monitoring, and research activities; the obtained results; the structure of project coordination; and the sectors involved in specific activities.

Objectives	%	n	Obtained Results	%	n
Acquire epidemiological data/clarify epidemiology	66.6	199	Report of pathogen prevalence in the study area/host species	64.5	193
Identification, serotyping, and genetical characterization	22.7	68	Confirm/discard endemicity or epidemiological role of a species	34.4	103
Collect data for risk assessment	13.7	41	Identify pathogens	22.1	66
Describe pathogenesis/histopathologic changes	13.4	40	Assess presence of zoonotic pathogens/contaminants in meat of game species	18.7	56
Data collection for surveillance	11.7	35	Describe pathogenesis/histopathological lesions	14.7	44
Antimicrobial resistance assessment	5.0	15	Recommend improvement of diagnostic techniques	5.4	16
Comparison of laboratory techniques	4.7	14	Detection of antimicrobial-resistant strains	4.3	13
**Project Coordination**	**%**	**n**	**Sectors Involved**	**%**	**n**
Academia	77.6	232	Veterinary sector	99.3	297
Public veterinary services	62.9	188	Ecology sector	8.7	26
Public human health services	12.7	38	Agro-forestal sector	7.7	23
Natural Parks	3.7	11	Human sector	7.4	22
			Biology sector	4.3	13
			Pharmaceutical	1.3	4

**Table 4:** The table shows the percentage and absolute number of bibliographical sources that detail the metadata collected for epidemiological purposes, sampled tissues and organs, and the type of diagnostic analysis. NUT2—Nomenclature of Territorial Units for Statistics-level 2; LAU2—Local Administrative Units level 2.

Metadata	%	n
Georeferencing	84.6	253
Anagraphical data	57.2	171
NUT3 level	34.4	103
Biometrical data	20.7	62
Hunting district	19.7	59
NUT2 level	12.0	36
LAU2 level	12.0	36
Coordinates	6.4	19
**Sampled Tissues/Organs**	**%**	**n**
Cardiac/abdominal blood cloth	27.4	82
Liver	21.4	64
Gastrointestinal tract	16.4	49
Feces	16.1	48
Spleen	15.7	47
Lungs	13.4	40
Skeletal muscle	12.7	38
Lymph nodes	11.7	35
Kidneys	11.4	34
Heart	9.0	27
**Diagnostics**	**%**	**n**
Molecular biology	60.2	180
Serology	28.8	86
Parasitological analysis	22.4	67
Bacteriological analysis	18.1	54
Macroscopic assessment	15.4	46
Microscopy	8.4	25
Chemical/physical analysis	5.4	16
Viral isolation	3.3	10

**Table 5:** Main bacteria, viruses, parasites, contaminants, and AMR strains evaluated in studies and surveillance plans.

Pathogens	%	n
**Bacteria**		
*Salmonella* spp.	6.7	20
*Brucella suis*	5.4	16
*Anaplasma phagocytophilum*	4.0	12
*Leptospira* spp.	4.0	12
*Yersinia enterocolitica*	3.7	11
*Mycobacterium tuberculosis* complex	3.3	10
**Virus**		
*Hepatitis E* virus	8.0	24
Aujeszky virus	4.0	12
West Nile	3.0	9
Usutu virus	3.0	9
*Porcine circovirus*	3.0	9
**Parasites**		
*Toxoplasma gondii*	8.7	26
Gastrointestinal helminths	8.7	26
*Trichinella* spp.	5.0	15
Broncho-pulmonary Nematodes	3.7	11
*Babesia* spp.	3.3	10
**Contaminants**		
Heavy metals	2.0	6
Ochratoxin A	1.3	4
Organochlorides and organophosphates	1.3	4
PCBs/PFAS/PBDEs	1.0	3
Cesium 137	0.7	2
**Antimicrobial-resistant strains**		
*E. coli*/*E. coli* β-lactamase-producing/*E. coli* colistin-resistant	1.7	5
*Salmonella* spp.	1.3	4
*Yersinia enterocolitica*	0.7	2
*Listeria monocytogenes*	0.3	1
Methicillin-Resistant *Staphylococcus aureus*	0.3	1

## Data Availability

All data are available within the manuscript or provided as Supplementary Materials.

## Supplementary Materials

The following supporting information can be downloaded at https://www.mdpi.com/article/10.3390/ani14152204/s1: Annex S1: bibliographic sources and data model; Annex S2: Pathogens targeted and their relative frequency.
