# Supplementary material for: The Role of Hunters in Wildlife Health Research and Monitoring: Their Contribution as Citizen Scientists in Italy
Source: Animals (Basel). 2024 Jul 29;14(15):2204. doi: 10.3390/ani14152204 (PMC11311091; doi:10.3390/ani14152204)
Supplement: Supplementary file 1 [file animals-14-02204-s001.zip › ANNEX S2.pdf]

Annex S2 Pathogens targeted by included surveillance systems and research studies and the relative frequency with whom they are targeted.

Target pathogen	n. of studies
Salmonella spp	20
Yersinia enterocolitica	11
Campylobacter spp	5
Escherichia coli spp.	9
Rickettsia SFG	9
Rickettsia slovaca	1
Borrelia burgdorferi s.l.	6
Brucella suis	16
Anaplasma phagocytophylum	12
Leptospira spp.	12
Staphylococcus aureus	3
My. Tubercolosis complex	10
MOTT	2
Ehrlichia spp	6
Listeria monocytogenes	5
Clostridium perfringens	2
Corynebacterium pseudotubercolosis	1
Coxiella burnettii	6
Francisella tularensis	5
Bartonella spp.	1
Aerobic colonies	2
Enterobacteriacae	3
Mycoplasma spp.	2
Mycoplasma conjunctivae	2
Pasteurella spp.	1
Erysipelothrix rhusiopathiae	1
Staphylococcus positive coagulase	1
Solphite-reducing clostridia	1
Treponema spp.	1
Clamidia spp.	3
Dermatophilus congolensis	2
Cilia-associated Bacillus	1
Lactic bacteria	1
Canine Distemper Virus	5
Porcine Circovirus	9
Torque Teno Suis Virus	1
Hepatitis E Virus	24
Hare Calicivirus	1
Mammalian Orthoreovirus	2
Avian Influenza	2
West Nile virus/ USUTU Virus	9

Orf Virus	1
Japanese Encephalitis Virus	1
Bluetongue Virus	1
Epiteliotrophic Virus	1
Schmallenberg Virus	1
Aujesky Virus	12
Canine Adenovirus	2
Rabies Virus	2
Porcine Respiratory Reproductive Syndrome Virus (PRRS)	1
Suid Herpesvirus	2
Porcine Parvocirus	2
Swine Vescicular Virus	2
Classical Swine Fever Virus	3
African Swine Fever Virus	2
Bovine Herpesvirus	1
Bovine Pestivirus	2
Bovine Parainflenza 3 Virus	1
Bovine Respiratory Syncytial Virus	1
Canine/Feline Parvovirus	2
Canine Circovirus	1
Aleutian Mink Disease Parvovirus	1
Porcine Pestivirus	1
Avipoxvirus	1
Ljungan Virus	1
Chronic Wasting disease	1
Ticks/flea	9
Lipoptena spp.	5
Angiostrogylus vasorum	6
Coccidia	6
Giardia spp	7
Cryptosporidium spp	5
Baylisascaris procionis	1
Gastrointestinal nemathods	25
Leishmania spp.	6
Babesia spp.	10
Encephalitozoon spp.	1
Hepatozoon spp	3
Echinococcus spp.	6
Blastocystis spp	1
Contracoecum rudolphii	2
Toxoplasma gondii	26
Trichinella spp	15
Theileria	2
Neospora caninum	6
Sarcocysitis spp.	3
Pulmonary nematods	11

Sarcoptes scabiei	3
Demodex spp.	1
Fasciola hepatica/Fascioloides/Dicrocoelium	4
Alaria alata	6
Trombicula autumnalis	1
Onchocerca jakutensis	1
Eucoleus garfiai	1
Fungi dermatophyti	1
Urinary nematods	2
Candida spp.	1
Capillaria hepatica	1
Haemoproteus/plasmodium	1
Leucocytozoon	1
Ochratoxin A	4
Cesium 137	2
Cadmium	3
Lead	3
Chromium	1
PCB	2
Organochlorine	3
Organophosporus	1
PFA/PBDE	1
Escherichia coli	2
Escherichia coli Beta lactamasi producing	1
Escherichia coli Colistin resistant	2
Salmonella	4
Yersinia enterocolitica	2
Listeria monocytogenes	1
Meticillin resistant *Staphylococcus aureus*	1
